# Zoledronic Acid Inhibits Lipopolysaccharide‐Induced Osteoclastogenesis by Suppressing Macrophage NLRP3‐Mediated Autophagy Pathway

**DOI:** 10.1002/iid3.70094

**Published:** 2024-12-16

**Authors:** Yuting Cheng, Guanjuan Liu, Xiaolin Huang, Yue Xiong, Na Song, Zheqing An, Wei Hong, Chidchanok Leethanakul, Bancha Samruajbenjakun, Jian Liao

**Affiliations:** ^1^ School/Hospital of Stomatology Guizhou Medical University Guiyang China; ^2^ Faculty of Dentistry Prince of Songkla University Hat Yai Thailand; ^3^ Hospital of Stomatology, Zhongshan City Zhongshan China; ^4^ Key Laboratory of Endemic and Ethnic Diseases Guizhou Medical University, Ministry of Education Guiyang China

**Keywords:** autophagy, bone resorption, lipopolysaccharide, NLRP3, osteoclast, zoledronic acid

## Abstract

**Introduction:**

Inflammatory factors leading to bone loss significantly increase the risk of tooth loosening or implantation failure. Zoledronic acid (ZOL) is a widely used medication for effectively inhibiting excessive bone destruction, but its effect on alleviating inflammatory bone loss remains to be elucidated. In this study, we investigated whether ZOL alleviates inflammatory bone resorption through immunomodulatory effect.

**Methods:**

The viability of the cells was evaluated by Cell Counting Kit 8 (CCK8) assay. Osteoclast (OC) differentiation and function were determined by tartrate‐resistant acid phosphatase (TRAP) staining and bone resorption pits assays, respectively. Autophagosomes and actin ring structures of OC were observed using transmission electron microscopy (TEM) and F‐actin ring staining, respectively. The microstructure in mice maxillary alveolar bone model was observed by micro computed tomography (Miro‐CT). Reverse transcription‐quantitative PCR (RT‐qPCR) to detect the mRNA expression of osteoclast‐related genes and Western blot (WB) analysis to evaluate the protein expression levels of autophagy‐related proteins and the NOD‐like receptor family pyrin domain‐containing protein 3 (NLRP3)‐related proteins in pre‐OCs.

**Results:**

The findings indicated that ZOL hindered lipopolysaccharide (LPS)‐mediated OC differentiation, formation, bone resorption activity and autophagosome levels. Furthermore, ZOL diminished the expression of genes associated with OC. And the expression of proteins ATG7, LC3II, Beclin1, NLRP3‐related proteins and tumor necrosis factor‐α (TNF‐α) protein were markedly decreased while P62 was increased, especially in the 1 μM ZOL group or MCC950 + ZOL group.

**Conclusions:**

ZOL has a certain immunomodulatory effect that exhibits anti‐inflammatory properties at lower concentrations, which can weaken LPS‐induced OCs differentiation and function, and NLRP3‐mediated autophagy pathway may participate in this process.

## Graphical abstract

This manuscript for the first time found that Zoledronic acid (ZOL) has a certain immunomodulatory effect that exhibits anti‐inflammatory properties at lower concentrations, which can weaken lipopolysaccharide (LPS)‐induced osteoclasts differentiation and function through inhibiting NLRP3‐autophagy pathway. These observations suggested that NLRP3/autophagy pathway have a great influence in the inhibition of osteoclastogenesis by ZOL in inflammatory microenvironment, and providing a new idea and a valuable reference for assessing the pharmacological impact of nitrogen‐containing bisphosphonates on bone tissue during inflammation and the application of this drug in the regulation of bone resorption in inflammatory bone diseases like periodontitis or peri‐implantitis.

## 1 Introduction

Chronic bone resorptive diseases, such as periodontitis or peri‐implantitis, cause disorders of homeostasis in the bone immune system [1, 2, 3]. Inflammatory bone loss around teeth or implants is a prominent risk factor for tooth loosening or implantation failure. Therefore, controlling inflammatory bone loss is crucial for the long‐term stability and survival rate of teeth or implant dentures [4]. A favorable local bone microenvironment facilitates effective osseointegration of the implant [5, 6]. Studies have revealed that inflammatory mediators and autophagy are associated with chronic bone resorptive disorders caused by trauma and inflammation, and are crucial for preserving the balance within the immune system of the bones [7, 8]. The NOD‐like receptor family pyrin domain‐containing protein 3 (NLRP3) inflammasome serves as the primary defense mechanism against pathogens. NLRP3‐mediated interleukin‐1β (IL‐1β) leads to inflammatory bone resorption and causes bone loss [7, 9]. In addition, by reorganizing the actin cytoskeleton, an increasingly active NLRP3 inflammasome can increase the process of bone resorption in osteoclasts (OCs) [10]. Therefore, elucidating the mechanisms of inflammatory bone resorption, as well as utilizing immune methods to regulate inflammatory mediators, is highly important for improving alveolar bone mass.

Zoledronic acid (ZOL), a highly effective medication that inhibits bone destruction and is a third‐generation nitrogen‐containing bisphosphonate, is the key to driving the osseointegration of implants because of its ability to greatly impact OCs [11, 12]. In our previous studies, we showed that ZOL could inhibit the activation of the receptor activator of nuclear kappa‐B ligand (RANKL)‐mediated nuclear factor kappa‐B (NF‐κB) signaling pathway [13, 14]. This interference effectively hindered the formation and bone resorption function of OCs and improved the degree of bone loss in rats with osteoporosis [15]. However, these studies focused on bone destruction alone rather than on inflammatory bone resorption. Moreover, most of the existing research has focused on high concentrations of ZOL to promote the development of inflammation, especially bisphosphonate‐related osteonecrosis of the jaw (BRONJ) [16, 17, 18]. Thus, we propose a research question: Does ZOL at low concentrations has a positive immunomodulatory effect on improving bone resorption in the inflammatory bone microenvironment. Some studies have reported that ZOL at extremely low concentrations has a positive effect on bone formation both in vitro and in vivo [19, 20, 21]. However, these previous studies were only preliminary explorations and did not involve corresponding molecular mechanism research. Actually, elucidating the mechanisms of inflammatory bone resorption and utilizing immune approaches to modulate inflammatory mediators are essential for improving alveolar bone mass. Therefore, the goal of our primary experiment was to clarify whether ZOL at low concentrations has a positive immunomodulatory effect and its molecular mechanism on improving bone resorption in the inflammatory bone microenvironment.

Autophagy can regulate the invasion of microbes and the secretion of immune signaling molecules and inflammatory mediators, thus playing a role in immunomodulation [22]. The activation of the NLRP3 inflammasome can modulate the initiation of autophagy, while autophagy plays a critical role in regulating the activation of the inflammasome and inhibiting its activity [23, 24]. In addition, studies have indicated that appropriate autophagy can suppress the activation of the NLRP3 inflammasome [25, 26]. The activation of the NLRP3 inflammasome triggers excessive OC autophagy, thereby resulting in an increase in both the quantity and activity of OCs, disrupting the balance of bone structure and function and ultimately leading to the inflammatory bone resorption [27, 28]. Therefore, the regulation of the autophagy/NLRP3 pathway is a potential method for the treatment of inflammatory bone diseases. Lipopolysaccharide (LPS), an essential inflammatory component found in the outer membrane of gram‐negative bacteria, can stimulate the generation of osteoclasts without relying on the presence of RANKL [29, 30]. Moreover, our preliminary studies demonstrated that OCs induced by LPS have actin‐ring structural activity and bone resorption functions and that ZOL has the capacity to suppress both the quantity and function of OCs stimulated by LPS. Based on the above evidence, we speculated that ZOL at low concentrations reduces autophagy level in macrophages by suppressing the NLRP3 pathway to downregulate IL‐1β, thereby preventing the formation of a large number of OCs and interfering with OCs function, which may be the molecular mechanism involved in alleviating inflammatory bone resorption.

## 2 Materials and Methods

### 2.1 Cell Culture and Animals

RAW264.7 cells (ATCC; Rockville, MD, USA) were cultured in α‒MEM (TFS Inc. USA) supplemented with a 1% solution of penicillin‒streptomycin and 10% fetal bovine serum (FBS, TFS Inc. USA) at 37°C in a 5% CO_2_ humidified environment, and the medium was replaced every 72 h. Twenty mice (8–10‐week‐old female C57BL/6J) were treated with LPS in phosphate‐buffered saline (PBS) (or with PBS as a vehicle) once a week (5 mg/kg, intraperitoneal) for 3 weeks as described previously [31]. Then, 20 μg/kg ZOL and MCC950 (10 μg/kg; Glpbio, CP‐456773, USA) were injected the day before LPS. The mice were killed via CO_2_ asphyxiation.

### 2.2 Cell Counting Kit 8 (CCK8) Assay

RAW264.7 cells were grown in 96‐well plates (5 × 10^3^ cells per well) and ZOL (0–30 µM) (Sigma‐Aldrich, Merck KGaA, Germany) together with 100 ng/mL LPS (Sigma‐Aldrich, Merck KGaA, USA) was added to the culture medium and further incubated for 24, 48 or 72 h. The supernatants were then removed, and 90 µL of newly prepared medium and 10 µL of CCK8 reagent (Dojindo Laboratories, Japan) were added. The plate was then incubated in total darkness at 37°C for an additional 3 h. The optical density at 450 nm was subsequently determined via an optical microplate reader (CMax Plus, USA).

### 2.3 In Vitro Osteoclastogenesis Assays

RAW264.7 cells were distributed in either 24‐well plates with 10^4^ cells per well for F‐actin ring staining or 96‐well plates with 1.5 × 10^3^ cells per well for tartrate‐resistant acid phosphatase (TRAP) staining and bone resorption pit assays. They were treated with various concentrations of ZOL for 1 h and then treated with LPS (100 ng/mL) for 3 days, 5 days or 10 days. The medium was replenished every 48 h.

#### 2.3.1 Tartrate‐Resistant Acid Phosphatase (TRAP) Staining

The medium was removed on the 3rd day. The cells were subsequently chemically fixed with 4% paraformaldehyde solution at 4°C for 20 min, followed by TRAP staining with a commercial kit (Solarbio, Beijing, China). This staining process took place at 37°C for 4 h in a light‐restricted environment. OCs exhibit dark red colouration and at least three nuclei were enumerated under a microscope (Leica, Germany).

#### 2.3.2 Bone Resorption Pit Assay

RAW264.7 cells were inoculated into 96‐well plates containing sterilized bovine bone slices (Millennium Biology, Shanghai, China). On the 10th day, the bone slices were washed with mechanical concussion and ultrasound to remove impurities and were gilded. A scanning electron microscope (Hitachi, Japan) was subsequently used to reveal the resorption pits.

#### 2.3.3 F‐Actin Ring Staining

RAW264.7 cells were seeded into 24‐well plates containing coverslips. On the 5th day, the cells were fixed at room temperature with 4% formaldehyde for 15 min. After the addition of 25% Triton X‐100, the sample was thoroughly rinsed with PBS. The F‐actin rings were labeled with TRITC rhodamine‐conjugated phalloidin (Solarbio, Beijing, China) for a 30‐min incubation period, while the cell nuclei were stained with DAPI (Solarbio, Beijing, China) for 30 s. Ultimately, the cells were examined via a confocal microscope (Olympus, Japan).

### 2.4 Transmission Electron Microscopy (Tem)

TEM (JEM‐1400FLASH, Japan) was used to inspect and identify the autophagosomes visually. Briefly, RAW264.7 cells (10^6^ cells per well) were cultured in 6‐well plates supplemented with 1 µM ZOL for 1 h and then treated with 100 ng/mL LPS. The cellular samples were collected, centrifuged, rinsed and treated with a solution containing 3% glutaraldehyde. The sample was subsequently fixed following immersion in a solution containing 1% osmium tetroxide, subjected to a sequence of acetone solutions for dehydration, immersed in Epox 812 for a prolonged period and embedded. The semithin sections were stained with methylene blue. After being sliced with a diamond knife, the ultrathin sections were then stained with uranyl acetate and lead citrate.

### 2.5 Micro Computed Tomography (Micro‐CT)

The microstructure in mice maxillary alveolar bone was scanned via high‐resolution Nemo Miro‐CT (NMC‐200, PINGSENG Healthcare Inc. Kunshan, China). The scanning parameters were 60 kV and 120 uA. The image was subsequently reconstructed via FDK 2 K on the Avatar software (2.0.11.0 PINGSENG Healthcare Inc). The pixel size is 8*8*9 µm, and the region of interest (ROI) is processed and analysed on the Avatar.

### 2.6 Reverse Transcription‒Quantitative Polymerase Chain Reaction (RT‒QPCR)

RAW264.7 cells (10^5^ cells per well) were seeded in 6‐well plates and stimulated with various concentrations of ZOL for 1 h. After the addition of 100 ng/mL LPS to the culture medium, the mixture was allowed to incubate for 12 h. Subsequently, TRIzol reagent (Invitrogen, USA) was used to extract total RNA. One microgram of total RNA was then used to synthesize complementary DNA (cDNA) with reverse transcriptase from the PrimeScript RT reagent kit (Takara Bio Inc, Japan). A SYBR Premix Ex Taq kit (TaKaRa Bio, Japan) and real‐time PCR detection system (Bio‐Rad, USA), known as CFX Connect, were used for RT‒qPCR. The PCR thermocycle conditions were as follows: precycling at 95°C for 30 s, followed by 39 cycles of denaturation (95°C, 5 s) and annealing (60°C, 30 s). The expression levels of the target genes were determined via comparison with the reference gene GAPDH via the 2^−ΔΔCT^ method [32]. The primer sequences (Sangon Biotech, Shanghai, China) utilized in this analysis can be found in Table 1.

**Table 1:** Sequences of quantitative PCR primers.

Primers Gene sequence
Mouse *TRAP* forward 5′‐ AAAGGGAGAGAACCAAATCC ‐3′
Mouse *TRAP* reverse 5′‐ ACAATACACCACCACATCCA ‐3′
Mouse *RANK* forward 5′‐TTCGACTGGTTCACTGCTCC‐3′
Mouse *RANK* reverse 5′‐TCAGGTGCTTTTCAGGGGAC‐3′
Mouse *NFATc1* forward 5′‐ TCCGTGTTCTGTCTGGTG ‐3′
Mouse *NFATc1* reverse 5′‐ CACTCATGTGCCCTGGA ‐3′
Mouse *GAPDH* forward 5′‐GGTTGTCTCCTGCGACTTCA‐3′
Mouse *GAPDH* reverse 5′‐TGGTCCAGGGTTTCTTACTCC‐3′

Abbreviations: NFATc1, recombinant nuclear factor of activated t‐cells, cytoplasmic 1; RANK, receptor activator of nuclear factor kappa B; TRAP, tartrate‐resistant acid phosphatase.

### 2.7 Western blot (WB) Analysis

RAW264.7 cells were cultured in a 6‐well plate with 1 × 10^6^ cells per well and were exposed to different concentrations of ZOL or 30 µM MCC950 (Glpbio, USA) for 1 h before being further stimulated with 100 ng/mL LPS for 6 h or 0, 6, 12 or 24 h. RIPA buffer (Solarbio, Beijing, China) was used to extract total protein from the cultured cells. The protein concentration was measured via a bicinchoninic acid (BCA) protein assay kit (Solarbio, Beijing, China). Equal amounts of protein (30 μg) from each sample were separated on 10% or 12% SDS polyacrylamide gels and transferred to PVDF membranes. The membranes were blocked with 5% skim milk for 2 h at room temperature and were incubated with anti‐NLRP3(1:1000, Cat#23094‐1), anti‐ATG7 (1:3000, Cat#6251), anti‐P62 (1:10000, Cat#4844), anti‐Beclin‐1(1:1000, Cat#19662), anti‐caspase‐1 (1:1000, Cat#16883), anti‐cleaved‐ caspase‐1 (1:1000, Cat#89332S), anti‐ tumor necrosis factor‐α (TNF‐α) (1:1000, Cat#19147), anti‐LC3II (1:1000, Cat#18709) (all from Abcam, USA), anti‐IL‐1β (Cell Signaling Technology, USA, Cat#12507) and β‐actin (PMK Biotechnology, Wuhan, China, Cat#083S) overnight at 4°C, followed by secondary antibodies (SAB, USA, Cat#L3012) at room temperature for a duration of 1.5 h. After being washed, an enhanced chemiluminescence (ECL) solution (Millipore, USA) was used for visualization of the membranes, and a gene gnome imaging system (Syngene, Europe) was used to identify the bands in the experiment.

### 2.8 Statistical Analysis

Statistical analysis was performed by GraphPad Prism 9.0 statistical software (San Diego, CA, USA). The Shapiro‒Wilk normality test was used to confirm the data to follow a normal distribution. Subsequently, multiple comparisons were carried out using a one‐way or two‐way ANOVA followed by Tukey's post‒hoc test. Each experiment was replicated three times, and all quantitative results are reported as the mean ± SD. Statistical significance was indicated by a *P* value of less than 0.05.

## 3 Results

### 3.1 Detection of the Viability of LPS‐Induced RAW264.7 Cells Treated With ZOL

**Figure 1:**
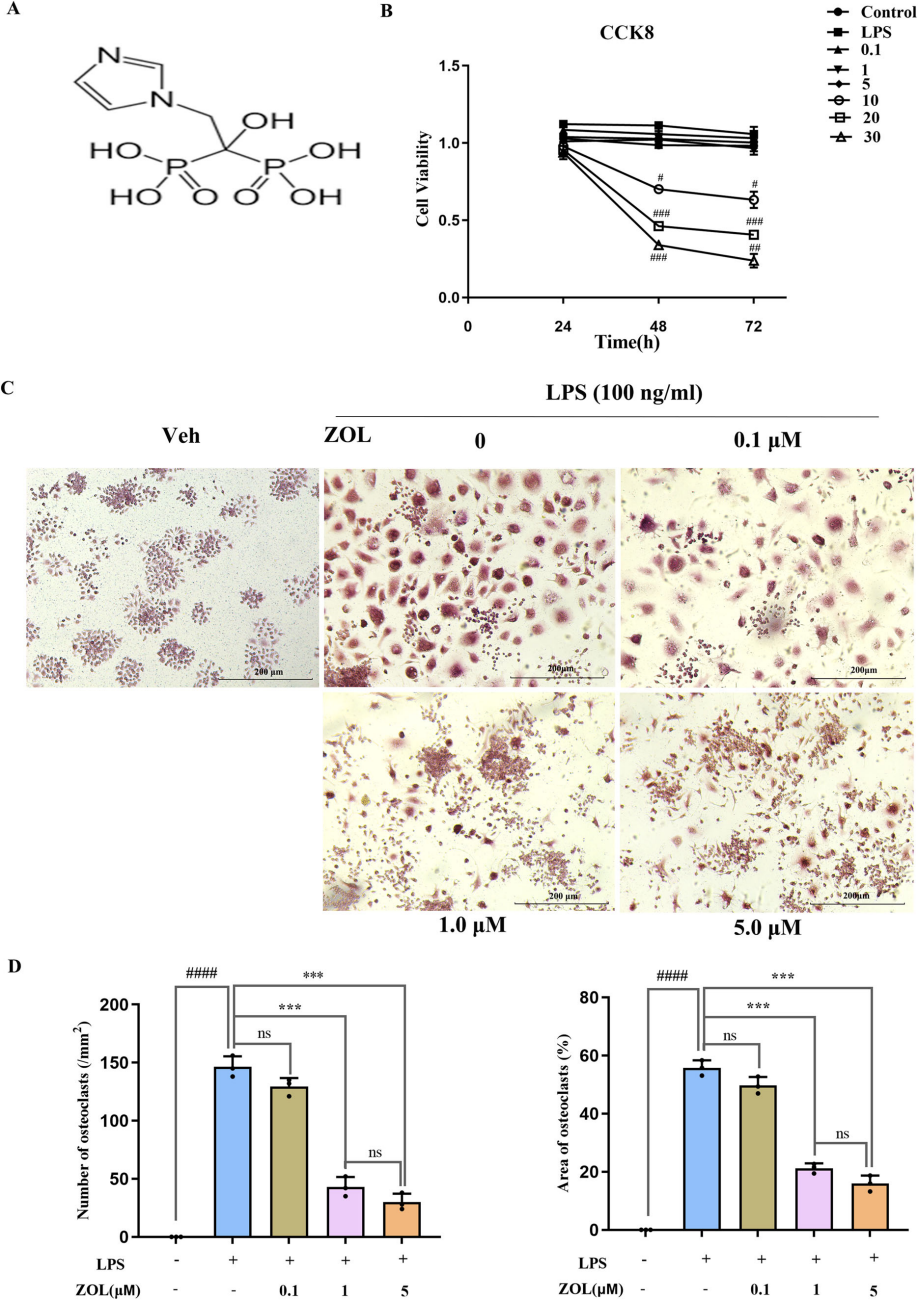
ZOL inhibited LPS‐induced TRAP multinucleated cell formation without cytotoxicity. (A) The chemical structure of ZOL. (B) RAW246.7 cells viability was detected by CCK‐8 assay after various time‐point (24, 48 or 72 h) in the presence of the indicated concentration of ZOL with 100 ng/ml LPS. (C) TRAP staining for the osteoclastogenesis assay, RAW264.7 cells were treated with 0.1,1, 5 µM ZOL L for 1 h and then treated them with 100 ng/mL LPS for 3 days. (D) The number and aera percentage of OCs were counted per field of microscope. Two‐way and one‐way ANOVA was employed for multiple comparisons, followed by Tukey's test for pairwise comparisons. Bars represent the mean ± SD, *n* = 3 independent experiments. ^*#*^ *p* < 0.05; ^*##*^ *p* < 0.01; ^*###* or ***^ *p* < 0.001; ^*####*^ *p* < 0.0001, ^*#*^ versus the control group or the vehicle group; ^*^versus the LPS‐treated group; ns, not significant. CCK‐8, cell counting kit 8; LPS, lipopolysaccharide; OCs, osteoclasts; TRAP, tartrate‐resistant acid phosphatase; ZOL, zoledronic acid.

The CCK8 assay data revealed significant suppression of cell growth by 10, 20 and 30 µM ZOL at times longer than 24 h (Figure 1B). Thus, 0.1, 1 and 5 µM ZOL were chosen for subsequent investigations to assess their potential inhibition of ability to inhibit LPS‐induced OCs.

### 3.2 ZOL Weakened LPS‐Induced Osteoclastogenesis

TRAP staining analysis revealed that LPS had triggered RAW264.7 cell transformation into OCs, and the fusion of multiple monocytes into multinucleated OCs was observed on the third day (Figure 1C). Conversely, the number of TRAP‐positive cells and the surface area taken up by OCs significantly decreased in the groups treated with either 1 or 5 µM ZOL (Figure 1C,D).

### 3.3 ZOL Inhibited LPS‐Stimulated Osteoclast Bone Resorption

**Figure 2:**
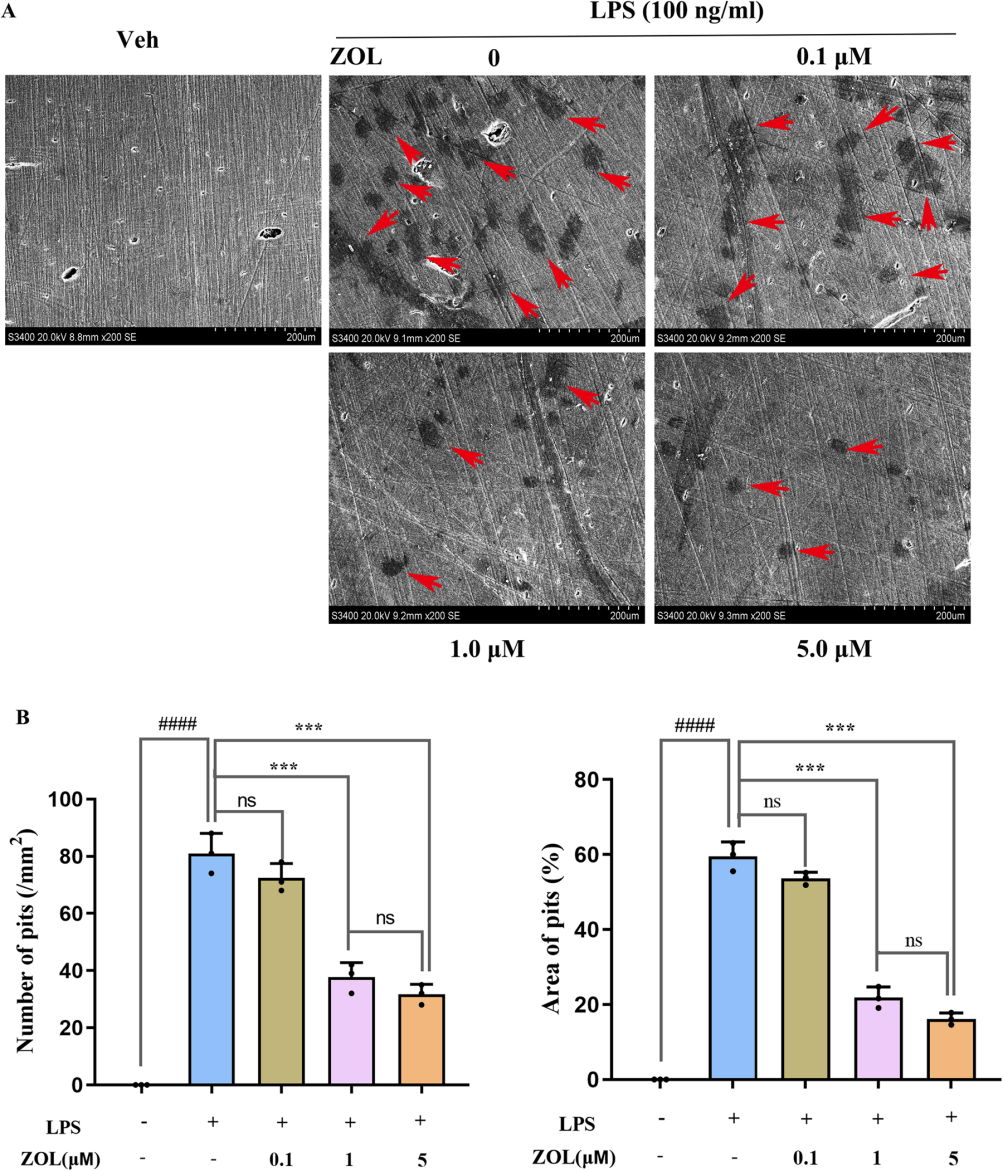
ZOL inhibited the LPS‐induced osteoclast bone resorption function. (A) Resorption pit assay was used to detect mature osteoclast activity. RAW264.7 cells were treated with various concentrations of ZOL for 1 h and then treated them with 100 ng/mL LPS for 10 days. Bone resorption pits (red arrows) were visualized under a scanning electron microscope. (B) The number and aera percentage of pits were quantified. One‐way ANOVA was employed for multiple comparisons, followed by Tukey's test for pairwise comparisons. Bars represent the mean ± SD, n = 3 independent experiments. ^*####*^ *p* < 0.0001; ^***^ *p* < 0.001, ^*#*^ versus the vehicle group; ^*^versus the LPS‐treated group; ns, not significant.

The results indicated that the area of the OC bone resorption pits was markedly decreased by ZOL in a dose‐dependent manner compared with that in the LPS group, and almost no resorption pits were observed in the groups treated with 5 µM ZOL (Figure 2A,B), indicating that ZOL treatment significantly impaired the bone‐resorption function of OCs.

### 3.4 ZOL Attenuated LPS‐Induced Changes in the Expression of Osteoclast‐Related Genes

**Figure 3:**
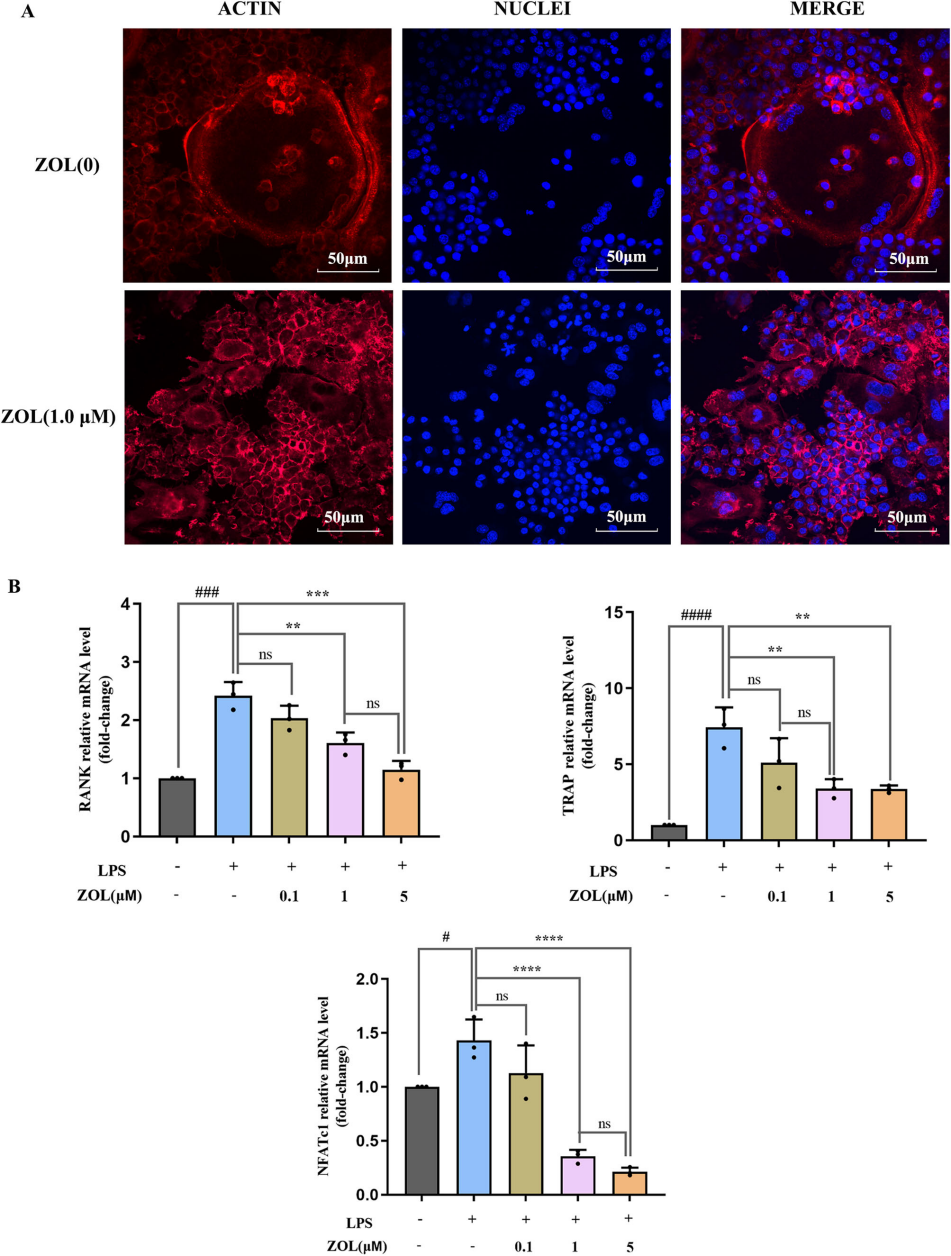
ZOL suppressed LPS‐induced osteoclast‐associated gene expression. (A) RAW264.7 cells were treated with ZOL (0 and 1 µM) for 1 h and then treated them with 100 ng/mL LPS for 5 days until mature osteoclasts were observed. Cell nuclei and F‐actin rings were stained with DAPI and TRITC phalloidin, respectively. (B) RAW 264.7 cells were stimulated with 0.1, 1 or 5 μM ZOL for 1 h and then treated them with 100 ng/mL LPS for 24 h, the expression of osteoclast‐specific genes (TRAP, RANK and NFATc1) were detected by RT‐qPCR. One‐way ANOVA was employed for multiple comparisons, followed by Tukey's test for pairwise comparisons. Bars represent the mean ± SD, *n* = 3 independent experiments. ^*#*^ *p* < 0.05; ^### or ***^ *p* < 0.001; ^*#### or* ****^ *p* < 0.0001; ^**^ *p* < 0.01, ^*#*^ versus the vehicle group; ^*^versus the LPS‐treated group; ns, not significant. NFATc1, recombinant nuclear factor of activated T‐cells, cytoplasmic 1; RANK, receptor activator of nuclear factor kappa B; RT‐qPCR, reverse transcription‑quantitative polymerase chain reaction.

As shown in Figure 3A, in the absence of ZOL (LPS group), there were large and intact actin ring structures, indicating that these mature OCs underwent bone resorption. Under the administration of 1 µM ZOL, the actin ring structure of OCs was destroyed, incomplete and deformed, and the shape decreased, revealing that mature OCs were blocked in the process of formation by ZOL. We subsequently studied the impact of ZOL on OC formation at the genetic level and assessed the mRNA expression levels of osteoclast‐associated genes induced by LPS after treatment with ZOL for 12 h.

The expression levels of TRAP, receptor activator of nuclear factor kappa B (RANK) and recombinant nuclear factor of activated T‐cell, Cytoplasmic 1(NFATc1) were markedly increased after induction with LPS. In contrast, the mRNA expression of these genes was significantly reduced by treatment with ZOL, and no substantial variation was detected in the outcomes of the 1 µM and 5 µM ZOL groups (Figure 3B).

### 3.5 ZOL Suppressed LPS‐Induced Autophagy by Inhibiting the NLRP3 Pathway in Pre‐OCS

**Figure 4:**
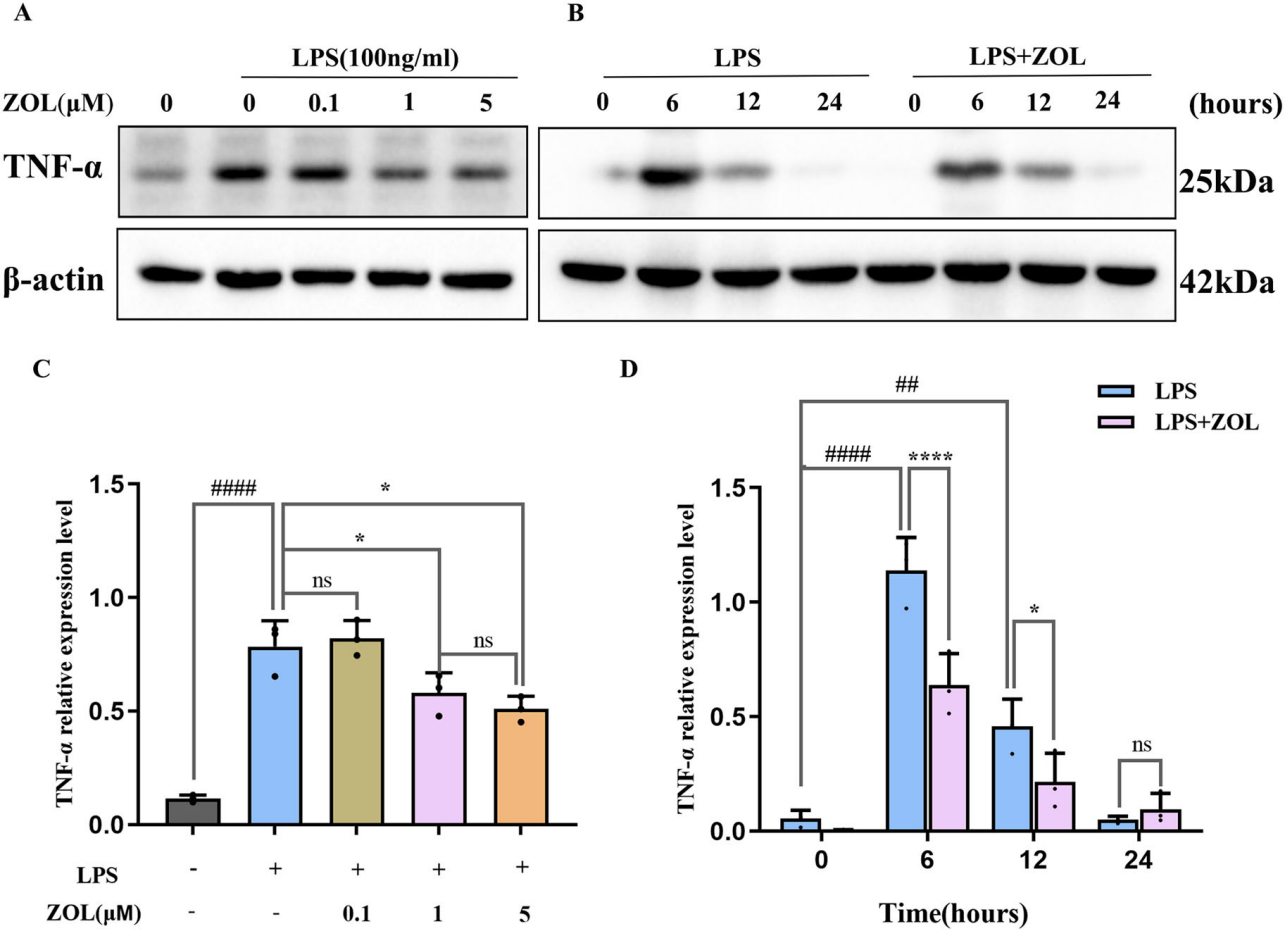
ZOL suppressed LPS‐induced inflammatory cytokine TNF‐α expression. To explore the peak time of inflammation induced by LPS, WB analysis was performed using TNF‐α antibody. RAW264.7 cells were cultured with 0.1, 1 or 5 μM ZOL for 1 h and then treated them with 100 ng/mL LPS for 6 h (A) and were induced with LPS for the indicated time points following pre‑treatment with ZOL (1 µM) for 1 h (B), (C). (D) The band intensities were quantified using Image J software. Two‐way ANOVA was employed for multiple comparisons, followed by Tukey's test for pairwise comparisons. Bars represent the mean ± SD, *n* = 3 independent experiments. ^*##*^ *p* < 0.01; ^*####* or ****^ *p* < 0.0001; ^*^ *p* < 0.05, ^*#*^ versus the vehicle group; ^*^versus the LPS‐treated group; ns, not significant. TNF‐α, tumor necrosis factor‐α; WB, western blot.

**Figure 5:**
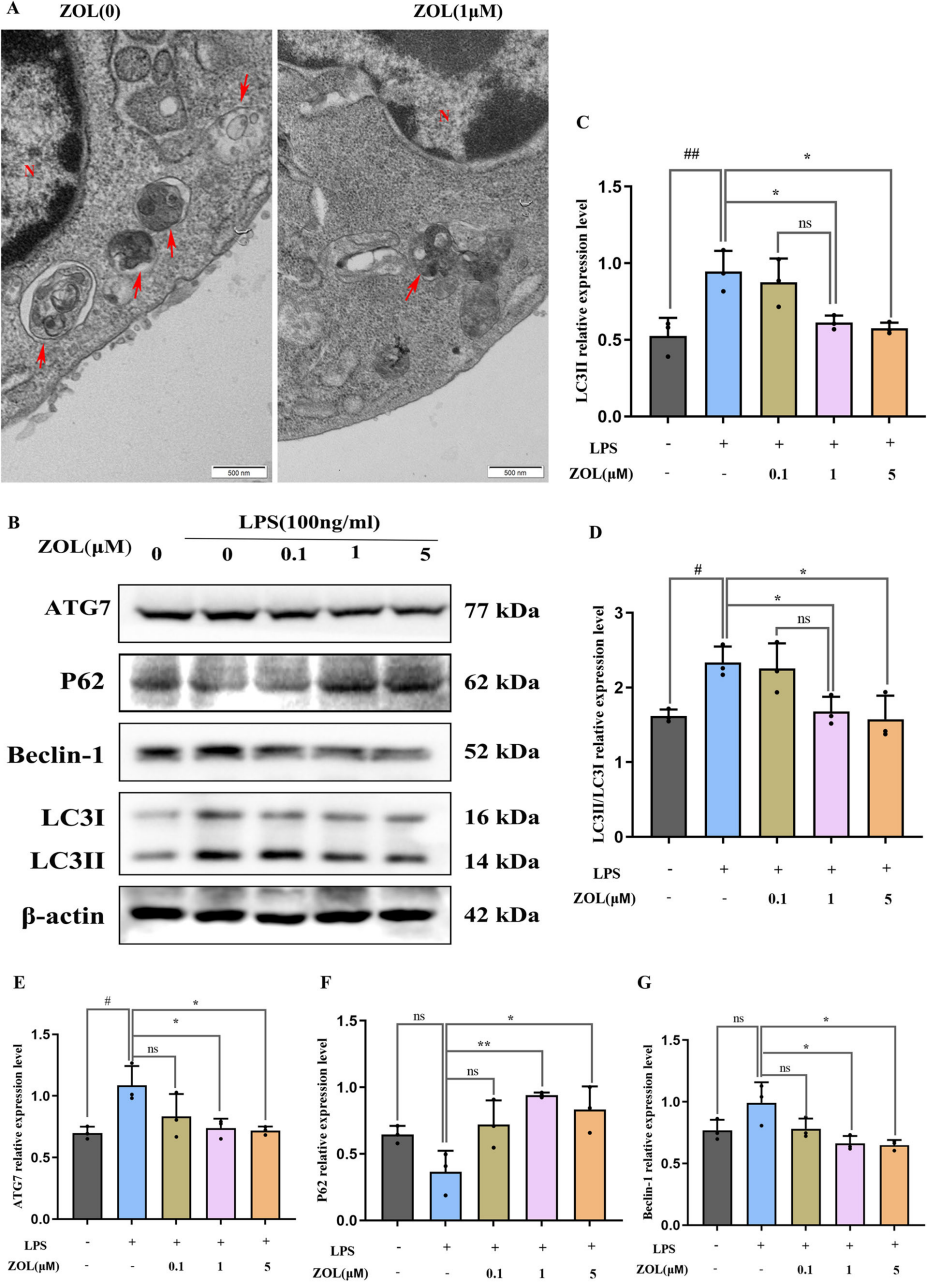
ZOL attenuated LPS‐induced autophagy in macrophages. (A) RAW264.7 cells were cultured with 100 ng/mL LPS for 12 h plus 1 μM ZOL, then autophagosomes (red arrow) as observed by TEM, N represents the nucleus. (B) RAW264.7 cells were incubated with 0.1, 1 or 5 μM ZOL for 1 h and then treated them with 100 ng/mL LPS for 6 h, then WB analysis was performed using autophagy‐related molecules. (C–G) The band intensities were quantified using Image J software. One‐way ANOVA was employed for multiple comparisons, followed by Tukey's test for pairwise comparisons. Bars represent the mean ± SD, *n* = 3 independent experiments. ^*#* or *^ *p* < 0.05; ^*##* or **^ *p* < 0.01, ^*#*^ versus the vehicle group; ^*^versus the LPS‐treated group; ns, not significant. TEM, transmission electron microscopy.

**Figure 6:**
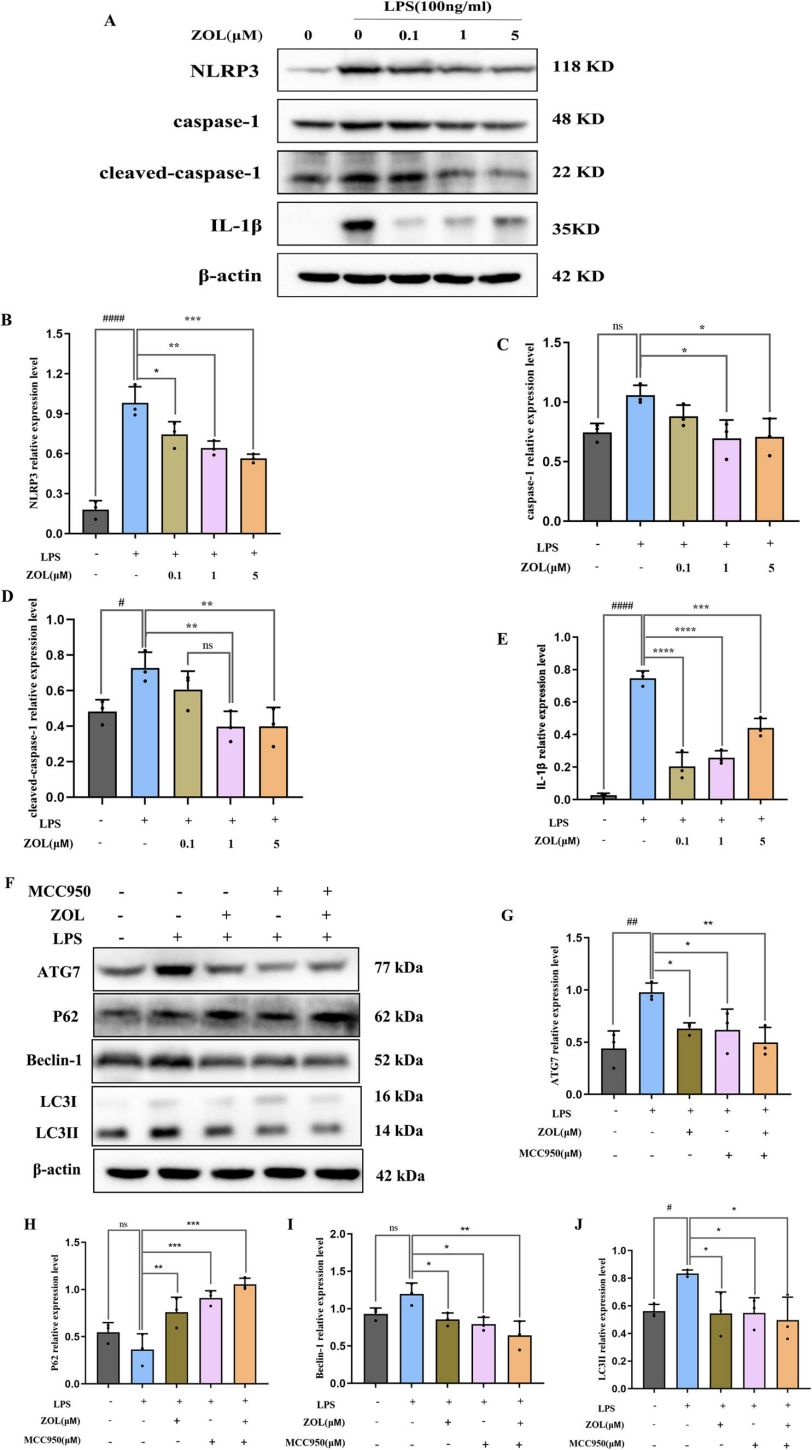
ZOL reduced autophagy by regulating NLRP3 pathway. (A) RAW264.7 cells were cultured with 0.1, 1 or 5 μM ZOL for 1 h and then treated them with 100 ng/mL of LPS for 6 h. (F) MCC950 (30 μM) and 1 µM ZOL were pretreated into LPS‐stimulated RAW264.7 cells for 6 h. (B–E) and (C–J) The band intensities were quantified using Image J software. One‐way ANOVA was employed for multiple comparisons, followed by Tukey's test for pairwise comparisons. Bars represent the mean ± SD, *n* = 3 independent experiments. ^# or *^ *p* < 0.05; ^*##* or **^ *p* < 0.01; ^***^ *p* < 0.001; ^*####* or ****^ *p* < 0.0001, ^*#*^ versus the vehicle group; ^*^versus the LPS‐treated group; ns, not significant.

First, we analysed the effect of the TNF‐α protein on inflammation (0, 6, 12 and 24 h), and found that the maximum inflammation induced by LPS occurred after 6 h (Figure 4B,D) and that ZOL inhibited the production of the pro‐inflammatory molecule TNF‐α, which is induced by LPS (Figure 4A,C). Additionally, compared with those in the LPS group, TEM analysis revealed a notable decrease in the number of autophagosomes in the group treated with ZOL (Figure 5A). Most importantly, after treatment with various doses of ZOL, the expression levels of proteins involved in autophagy and NLRP3 changed significantly (Figure 5B and Figure 6A). Compared with those in the LPS group, the levels of LC3II, Beclin1, ATG7, NLRP3, cleaved‐caspase‐1 and IL‐1β were markedly lower in the groups treated with 1 µM and 5 µM ZOL, followed by a notable increase in the p62 protein expression level (Figure 5C‐G and Figure 6B–E). The expression of autophagy‐related proteins was subsequently suppressed by MCC950 (Figure 6F), especially in the ZOL + MCC950 group, and no considerable difference was observed between the MCC950 group and the ZOL group (Figure 6G–J).

### 3.6 Effect of ZOL on the Microstructure of Maxillary Alveolar Bone

**Figure 7:**
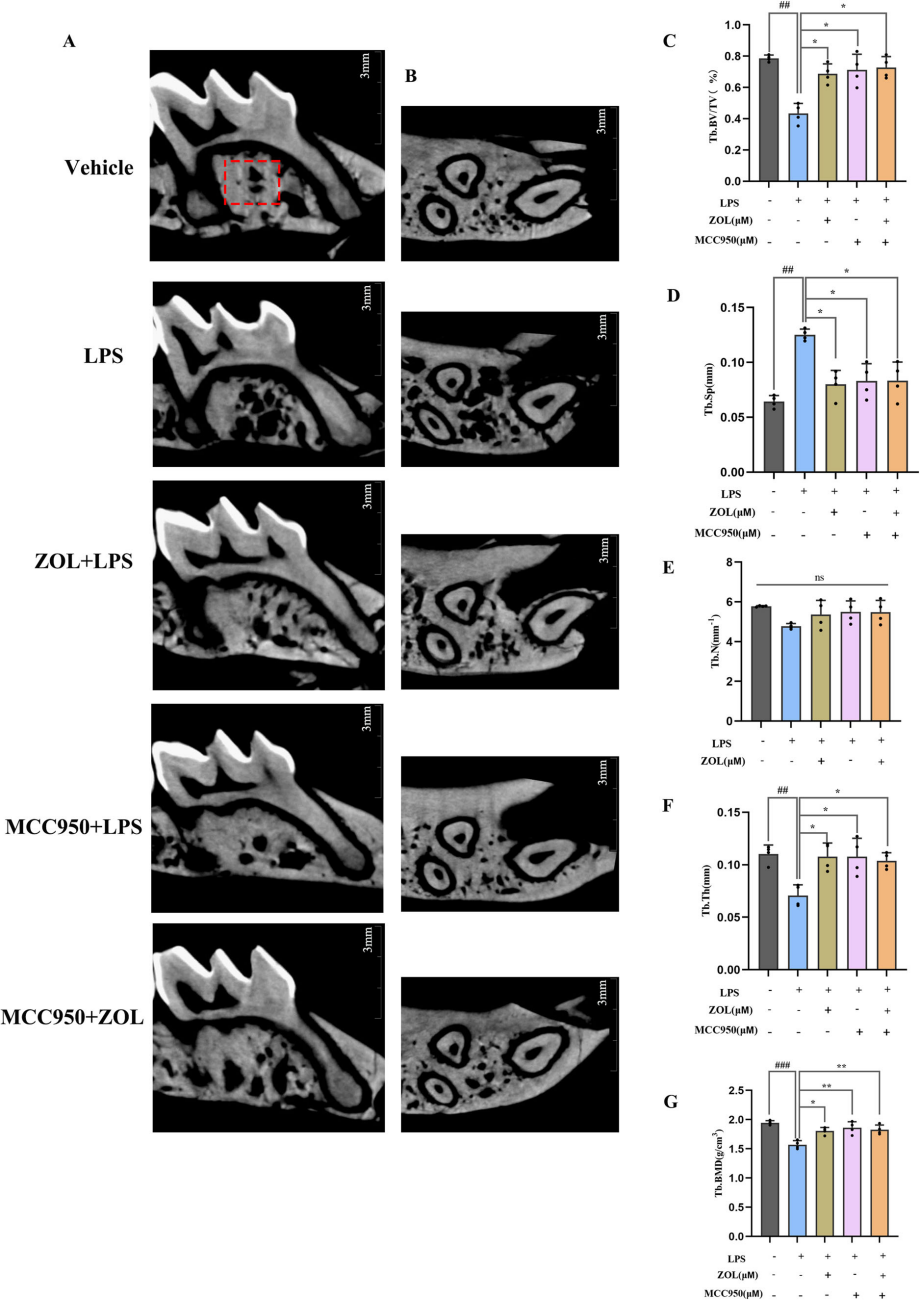
Miro‐CT in the maxillary alveolar bone. (A) Coronal Miro‐CT images of mouse maxillary alveolar bone; (B) Cross‐sectional Miro‐CT images of mouse maxillary alveolar bone, (C) BV/TV (%), (D) Tb. Sp (mm); (E) Tb. N (mm^−1^); (F) Tb. Th (mm); (G) Tb. BMD (g/cm^3^). One‐way ANOVA was employed for multiple comparisons, followed by Tukey's test for pairwise comparisons. Bars represent the mean ± SD, n = 4 independent mice. ^*##* or **^ *p* < 0.01; ^*###*^ *p* < 0.001; ^*^ *p* < 0.05, ^*#*^ versus the vehicle group; ^*^versus the LPS‐treated group; ns, not significant. BV/TV, bone volume/total volume; Tb. Sp, trabecular space; Tb. Th, trabecular thickness; Tb. BMD, trabecular bone mineral density.

Miro‐CT revealed that the trabeculae of the maxillary alveolar bone in the vehicle group were dense and neatly arranged, whereas in the LPS group, the bone mass was lost, the trabeculae became narrower, and the degree of interosseous dispersion of the trabeculae increased (Figure 7A,B). The analysis of microstructural parameters of alveolar bone revealed that, compared with those in the vehicle group, the bone volume/total volume (BV/TV), trabecular thickness (Tb. Th), and the trabecular bone mineral density (BMD) decreased and the trabecular space (Tb. Sp) increased in the LPS group; however, after treatment with ZOL and MCC950, the alveolar bone mass increased, the trabecular thickness increased, the degree of bone dispersion decreased and the degree of bone arrangement was dense (*p* < 0.05), but there was no significant difference in the number of trabeculae among all the groups (*p* > 0.05) (Figure 7C‒G).

## 4 Discussion

In the present study, we discovered that ZOL has an anti‐inflammatory effect within a range of low concentrations and has the ability to inhibit inflammatory osteoclastogenesis, which is closely related to the NLRP3‐mediated autophagy pathway. Inflammatory bone loss around the teeth or the dental implants is a crucial element that impacts tooth loosening or implantation failure [2]. ZOL is widely used to treat bone resorption, and can effectively promote the osseointegration of implants, mainly by affecting OCs [11, 12]. Our previous studies focused only on bone destruction alone rather than on inflammatory bone resorption [13, 14, 15]. In addition, existing studies have focused on investigating the impact of high levels of ZOL on the progression of LPS‐induced inflammation [16, 17], and only a few have studies suggested that ZOL at low concentrations has a positive effect on bone formation [20, 21]. However, the mechanism involved in these findings is relatively superficial. Our study revealed that ZOL not only has anti‐inflammatory properties at relatively low concentrations, but also further explored the corresponding mechanism. Notably, we observed that the dominant action of ZOL appears to be the suppression of pro‐inflammatory factor expression, excluding the production of anti‐inflammatory factors.

Many studies indicate that the NLRP3 inflammasome has the ability to regulate autophagy initiation, and that autophagy has the ability to control the activation of inflammasomes and impede their activity [23, 33, 34, 35]. Moreover, according to previous research, a possible approach for treating inflammatory bone loss could be to control the regulation of the LPS‐induced autophagy of OCs, which may help decrease the number and activity of OCs [36]. Therefore, the impact of ZOL on NLRP3 and autophagy activated by LPS deserves further investigation based on these findings.

OCs generated by RANKL are frequently employed as models for alone bone destruction disorders alone, whereas OCs triggered by LPS are commonly utilized as models to simulate chronic bone infections. Previous studies have shown that LPS‐stimulated macrophages can fuse without the assistance of other cells and form multinucleated OCs in a RANKL‐independent manner [37, 38, 39]. Our results, which included TRAP staining, F‐actin ring staining and bone resorption pit assays, also revealed that LPS‐induced multinucleated OCs were strongly capable of facilitating bone decomposition. Our findings concerning the TNF‐α protein (Figure 4) aligned with those of prior research [40], indicating that a duration of 6 h of LPS‐mediated inflammation resulted in the most significant alteration in the protein levels associated with it. Additionally, our animal model also showed a certain amount of bone mass loss (Figure 7). Taken together, the above results showed that LPS successfully induced inflammatory bone resorption. However, there was no significant change in the number of trabeculae, which may be related to the shorter duration of inflammation induction.

However, after the administration of 1 µM ZOL, the changes in the morphology of the cells were reversed. Furthermore, the mRNA expression levels of genes related to OC formation stimulated by LPS were assessed, and TRAP, RANK, and NFATc1 were significantly increased. RANK and NFATc1 are proteins involved in gene expression and are important for immune responses, which are thought to stimulate the growth and development of OCs, subsequently leading to inflammatory bone resorption [41, 42, 43]. However, the expression of these genes was noticeably inhibited after ZOL was administered. These findings suggest that this medication interferes with the maturation and functionality of LPS‐generated OCs and indirectly reflects that ZOL has certain anti‐ inflammatory properties.

Finally, we examined how ZOL affects autophagy and the NLRP3 pathway at the protein level (Figure 5 and Figure 6). After exposure to LPS for 6 h, the protein levels of NLRP3, cleaved‐caspase‐1, IL‐1β, LC3II and ATG7 significantly increased, indicating activation of the LPS‐induced NLRP3‐autophagy pathway. Previous studies have shown that activating the autophagy pathway by LPS can lead to an increase in the quantity and functionality of OCs during inflammatory bone loss [34, 44], suggesting that excessive autophagy can contribute to inflammation. The engagement of autophagy components, including ATG7, Beclin1, ATG5, and LC3, during the process of bone remodeling can be enhanced to facilitate OC formation at folded edges and accelerate the progression of bone resorption [45]. However, compared with the LPS group, ZOL actively suppressed these effects. These results suggest that ZOL hinders the activity of autophagy‐associated proteins in macrophages that are responsible for bone resorption, which has the potential to be a valuable strategy in the treatment and/or prevention of diseases related to OCs. Conversely, this discovery contradicts earlier research demonstrating that autophagy is a crucial factor for enhancing bone formation in individuals with osteoporosis [46, 47], which may be due to the role of autophagy in the relationship between different types of cells in different osteoporotic pathogeneses (estrogen, glucocorticoid, senescence and inflammation) [48, 49, 50]. Inflammation mainly enhances autophagy to participate in OC‐mediated bone resorption. The first three factors are mainly aimed at reducing the autophagy of OBs, bone marrow mesenchymal stem cells (BMSCs) and osteocytes (OSTs) rather than OCs. Additionally, there were no significant differences in the protein expression of P62 and Beclin‐1 between the vehicle group and the LPS group (Figure 5F,G and Figure 6H,I), which may be attributed to the 4‐h starvation period before drug administration. As a consequence, the levels of specific autophagy proteins tended to increase in some vehicle groups. Nevertheless, there were no notable alterations observed in the other autophagy proteins in the vehicle group, which could be linked to the brief period of starvation. The NLRP3 inflammasome promotes the secretion of pro‐inflammatory cytokines, which are responsible for bone resorption and cause the destruction of alveolar bone destruction [51], including IL‐1β and TNF‐α, which are known to promote bone loss during inflammatory infections [52, 53]. The expression of the ASC protein was not obvious in this study. Studies have confirmed that the relative ratio of ASC to caspase‐1 in cells is estimated to be 1:3.5 based on quantitative Western blot analysis, whereas this ratio does not seem to affect inflammasome activation [54, 55]. MCC950, a specific inhibitor of NLRP3, was subsequently used to further explore the underlying molecular mechanism. When MCC950 binds to NLRP3, it can be locked in the inactive domain to prevent NLRP3 aggregation and activation [56]. Notably, when the NLRP3 pathway was blocked with MCC950, the expression of autophagy‐related proteins was inhibited more significantly in the MCC950 + ZOL group than in the other groups, indicating a synergistic effect between ZOL and MCC950. Similarly, there was no distinct difference in the Miro‐CT results between the ZOL group and the MCC950 group (Figure 7), suggesting that ZOL at a low dose ( ≤ 0.04 mg/kg) relieved the development of inflammatory bone resorption [21].

However, although our findings indicate that ZOL reduces the expression levels of autophagy‐related proteins by suppressing NLRP3 signaling pathway, the current study has several limitations. First, we used simple culture induction of RAW264.7 macrophages instead of coculture with MC3T3 embryonic OBs; therefore, these studies did not clarify the effect of ZOL on bone formation under the influence of LPS, and the interactions among ZOL, LPS and autophagy on osteoblastogenesis remain largely unknown. In our investigation, we observed an absence of THP‐1 cells produced from human mononuclear macrophage leukemia cells. These cells are more specific and closely associated with clinical applications. Then, our animal experiments should not only use female mice. It will be more common if there is no gender difference. Finally, the findings of the present study must be validated by additional assays. For example, an overexpression viral vector and a knockout gene animal model could be used to further determine the associations among ZOL, autophagy and NLRP3.

**Figure 8:**
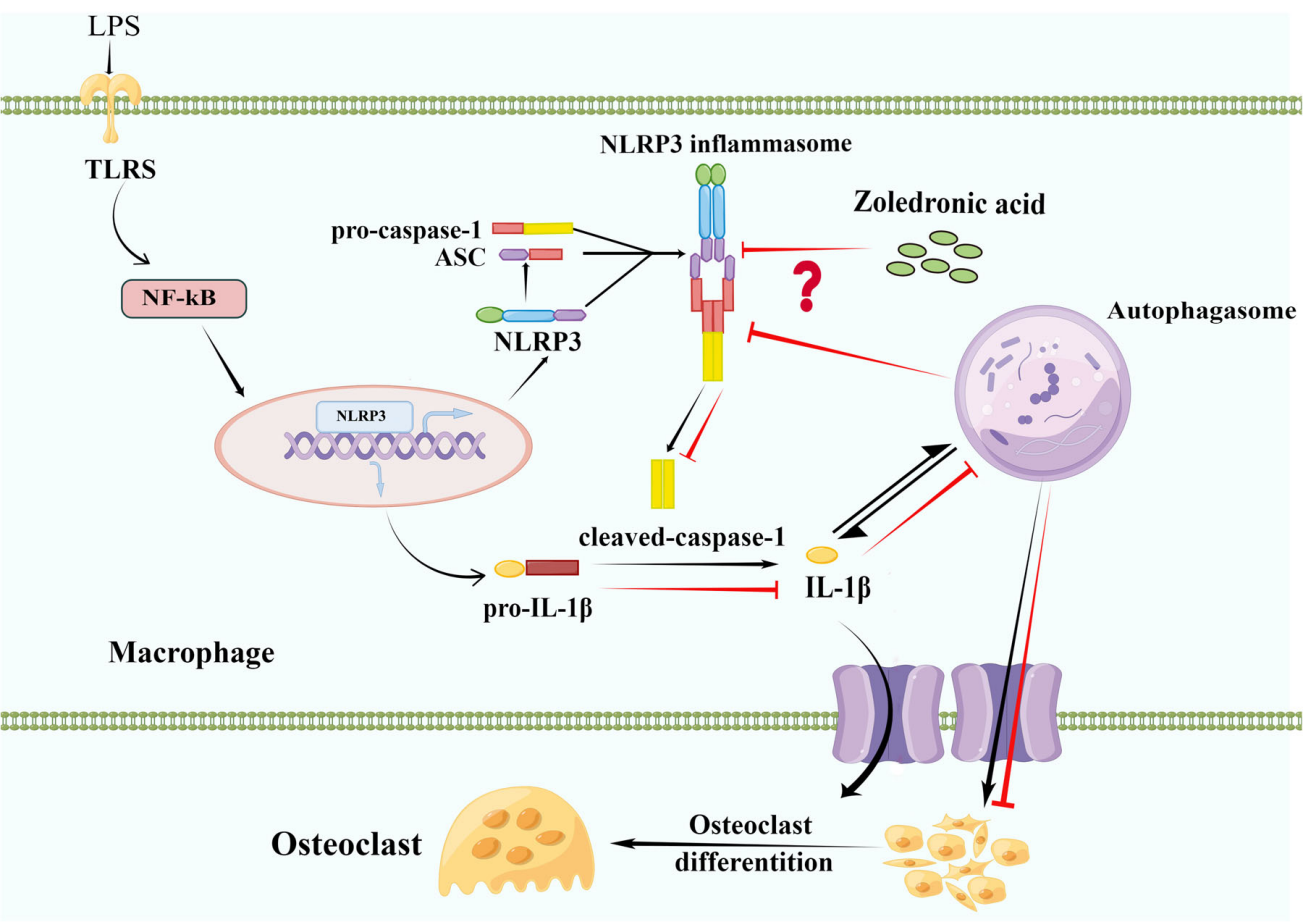
The mechanism of ZOL relieving inflammatory bone loss by reducing autophagy level through suppressing NLRP3 signaling pathway to downregulate IL‐1β. The NF‐κB signaling pathway is activated by the TLR pathway and further stimulates NLRP3 inflammasome assembly and activation. Then the precursor caspase‐1 into an activated form (cleaved‐caspase‐1). Activated caspase‐1 cleaves the precursor IL‐1β into mature forms and cause inflammation. In the inflammatory bone microenvironment, the NLRP3 inflammasome promotes autophagosomes greatly increased, and excessive autophagy aggravates the development of inflammation as well. However, ZOL may inhibit the activation of the NLRP3 inflammasome and downregulate IL‐1β to reduce the level of autophagy in macrophages, thus inhibiting the activity and function of OCs and alleviating inflammatory bone resorption. IL‐1β, Interleukin‐1β.

In conclusion, the results of the present study demonstrated that ZOL has certain immunomodulatory effects that exhibit anti‐inflammatory properties at lower concentrations; these effects can weaken LPS‐induced OC differentiation and function through the inhibition of the NLRP3‐mediated IL‐1β pathway to reduce overactivated autophagy (Figure 8). Our study could serve as a valuable reference for assessing the pharmacological impact of nitrogen‐containing bisphosphonates on bone tissue during inflammation, and provide insights into the use of these medications for the management of bone resorption disorders in patients with inflammatory bone diseases. Our prospect for future work mainly targets local inflammatory bone resorption without systemic bone disease, such as periodontitis or peri‐implantitis. Owing to its specific immunomodulatory impact and bone targeting characteristics can further alleviate the development of inflammatory bone resorption. Therefore, ZOL may be utilized as a adjunctive therapy following mechanical debridement of inflammatory bone lesions in the future.

## Author Contributions

**Yuting Cheng:** methodology, writing–original draft, investigation, formal analysis, data curation. **Guanjuan Liu:** methodology, data curation. **Xiaolin Huang:** methodology, data curation. **Yue Xiong:** investigation, writing–review and editing. **Na Song:** methodology, investigation. **Zheqing An:** methodology, investigation. **Wei Hong:** supervision, project administration. **Chidchanok Leethanakul:** Supervision, writing–review and editing. **Bancha Samruajbenjakun:** supervision, writing–review and editing. **Jian Liao:** conceptualization, supervision, formal analysis, funding acquisition.

## Ethics Statement

The animal study was approved by the Animal Experimental Ethics Committee of Guizhou Medical University (no. 2201593). All experimental procedures were performed in accordance with the guidelines of the International Council on Research Animal Care as well as the “Guide for the Care and Use of Laboratory Animals” by the National Institute of Health of the United States.

## Conflicts of Interest

The authors declare no conflicts of interest.

## Data Availability

The datasets generated during and analysed during the current study are available from the corresponding author on reasonable request.
